# Supplementary material for: 3D printing of ultra-high viscosity resin by a linear scan-based vat photopolymerization system
Source: Nat Commun. 2023 Jul 18;14:4303. doi: 10.1038/s41467-023-39913-4 (PMC10353997; doi:10.1038/s41467-023-39913-4)
Supplement: Supplementary file 1 — Supplementary Information [file 41467_2023_39913_MOESM1_ESM.pdf]

Supplementary Information

for

3D printing of ultra-high viscosity resin by a linear scan-based vat photopolymerization system

Zixiang Weng[1,2*], Xianmei Huang[1], Shuqiang Peng[1,3], Longhui Zheng[1], Lixin Wu[1,2*]

[1] CAS Key Laboratory of Design and Assembly of Functional Nanostructures, Fujian Key Laboratory of Nanomaterials, Fujian Institute of Research on the Structure of Matter, Chinese Academy of Sciences, Fuzhou 350002, P. R. China

[2] Fujian Science & Technology Innovation Laboratory for Optoelectronic Information of China, Fuzhou, Fujian 350108, P. R. China

[3] Key Laboratory of Polymer Materials and Products, College of Materials Science and Engineering, Fujian University of Technology, Fuzhou 350118, P. R. China

*Corresponding author.

E-mail: wzx@fjirsm.ac.cn (Zixiang Weng); lxwu@fjirsm.ac.cn (Lixin Wu)

Code of .bat file

The code of the .bat file for format conversion is as follows:

Box S1 | .bat file information

```
        @echo off
        cd /d%~sdp0
        echo%cd%
        set filepath=%1
        echo "%cd%\running\nanoslicer.exe"
        echo%filepath%
        rem step. 1 ================Slicing File================
        call "%cd%\running\nanoslicer\nanoslicer.exe" -f%filepath% -w 2100 -e 1800
-r 66.66667 -t 0.1
        rem 2100 and 1800 represent the resolution for the image.
        rem step. 2 ================Unzip sliced pictures================
        call "%cd%\running\WinRAR\WinRAR.exe" x -ad frames.zip % step 1%
        rem step. 3 ===========Transfer .png file to .bmp file===========
        call "%cd%\running\magick\magick.exe" mogrify -format bmp -rotate 270 -
type Bilevel frames\*.png
        @ping 127.0.0.1 -n 20 >nul
        del frames.zip
        del frames\*.png
pause
```

A created folder named as <frames> can be renamed and be recognized by the 3D printer through a SD card reader.

2. Comparison of Printing Speed between CLIP and LSVP technology

For CLIP technology: Because the process of resin leveling from the surroundings to the center forming position is regarded as the flow process in the tube, and it is a laminar flow of a non-circular tube, it is necessary to calculate the corresponding hydraulic diameter of the process according to **Eq. 1**:

$$D = \frac{4A}{p} = \frac{4 \times h \times b}{2 \times b} = 2h \quad (1)$$

where A represents the cross-sectional area of resin flow (mm^2); p represents peripheral length of solid in contact with fluid (mm); h represents the layer thickness (mm); b represents

the width of the resin flow (mm). According to the flow equation in the tube, considering the symmetry of the flow process, the average flow velocity during the flow in the tube is calculated according to **Eq. 2**:

$$V_{AV} = \frac{\Delta P D^2}{32\mu L} = \frac{\Delta P h^2}{4\mu a} \quad (2)$$

where *a* represents the distance of the resin flow (mm); Δ*P* represents the pressure difference flowing in the pipe; *μ* represents the apparent viscosity of the resin.

According to the average speed, the flow distance *l* of the resin when the molding platform drops at a constant speed at the speed *v* can be obtained from **Eq. 3**:

$$l = \int_0^t V_{\mathrm{AV}} dt = \int_0^t \frac{\Delta P h^2}{4\mu a} dt = \int_0^t \frac{\Delta P (vt)^2}{4\mu a} \cdot \int_0^t t^2\, dt = \frac{\Delta P \cdot v^2 \cdot t^3}{12\mu a} \quad (3)$$

Considering the resin was replenished from surrounds, we can assume *l*=*a*/4 and obtain:

$$t = \sqrt[3]{\frac{3\mu a^2}{\Delta P \cdot v^2}} \quad (4)$$

In **Eq. 4**, *t* represents the time for self-levelling, which is closely related to the viscosity of the resin. It increases with the increase of the resin.

For LSVP showed in this work. In this LSVP system, the printing speed is only related to the movement speed. The exposure energy is proportional to the number of scanning time. If the number of scanning time is 1, the movement of the laser modulus is 35 mm/s. For those resin with low reactivity, increase the number of scanning time will emit higher energy for curing. By increasing the number of scanning time to *x*, the movement of the laser modulus *v*=35/*x* cm s^{-1}. Hence the printing time is only related to the length in x axis and the reactivity of the UV-curable resin.

Table S1. Commonly used UV curable resin for 3D printing with low viscosity

Name	Structure/Brand	Functionality	Mass ratio
Aliphatic polyurethane acrylate	DSM NeoRad™ U-25-20D	Oligomer	20%
Bisphenol A epoxy acrylate	Sartomer CN104NS	Oligomer	20%
Acryloyl morpholine	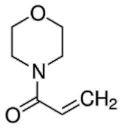	Monomer	25%
Ethoxylated trimethylolpropane triacrylate	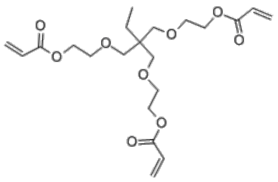	Monomer	27%
Dipropylene glycol diacrylate	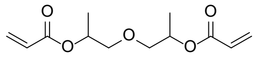	Monomer	5%
TPO	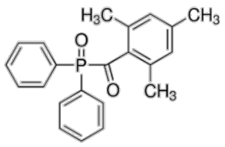	Photoinitiator	3%
White color paste	-	Additive	+0.1%
Fumed nanosilica	-	Additive	+0.2%

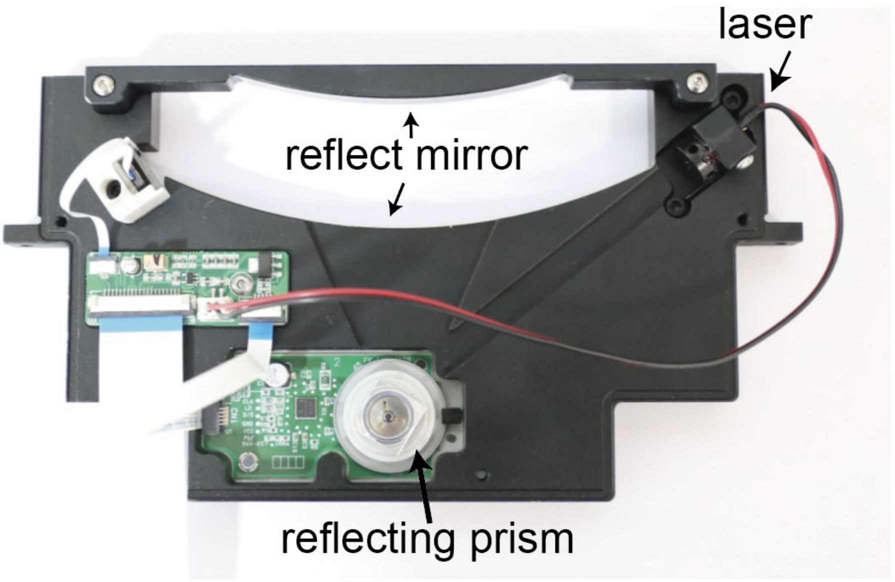

Fig. S1. Physical graph of laser module.

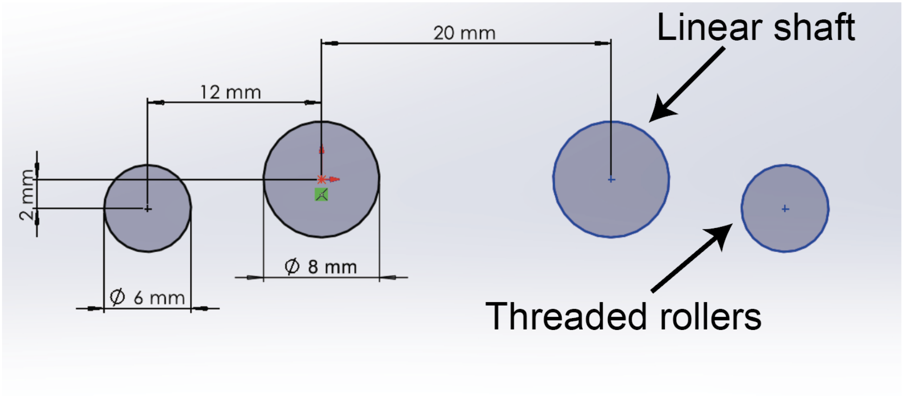

Fig. S2 Engineering drawing of four rollers.

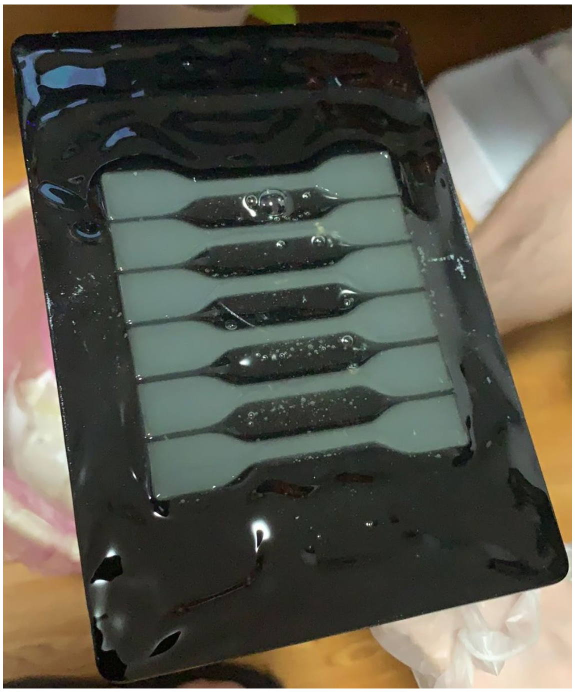

Fig. S3 Printed samples (by high viscosity resin) for tensile strength tests.

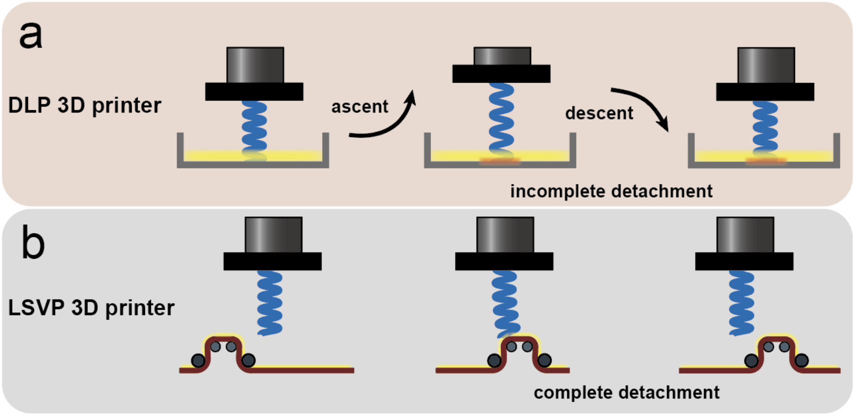

Fig. S4 Detachment fashion comparison between a DLP 3D printer and LSVP 3D printer **b** when fabricating elastomer material.

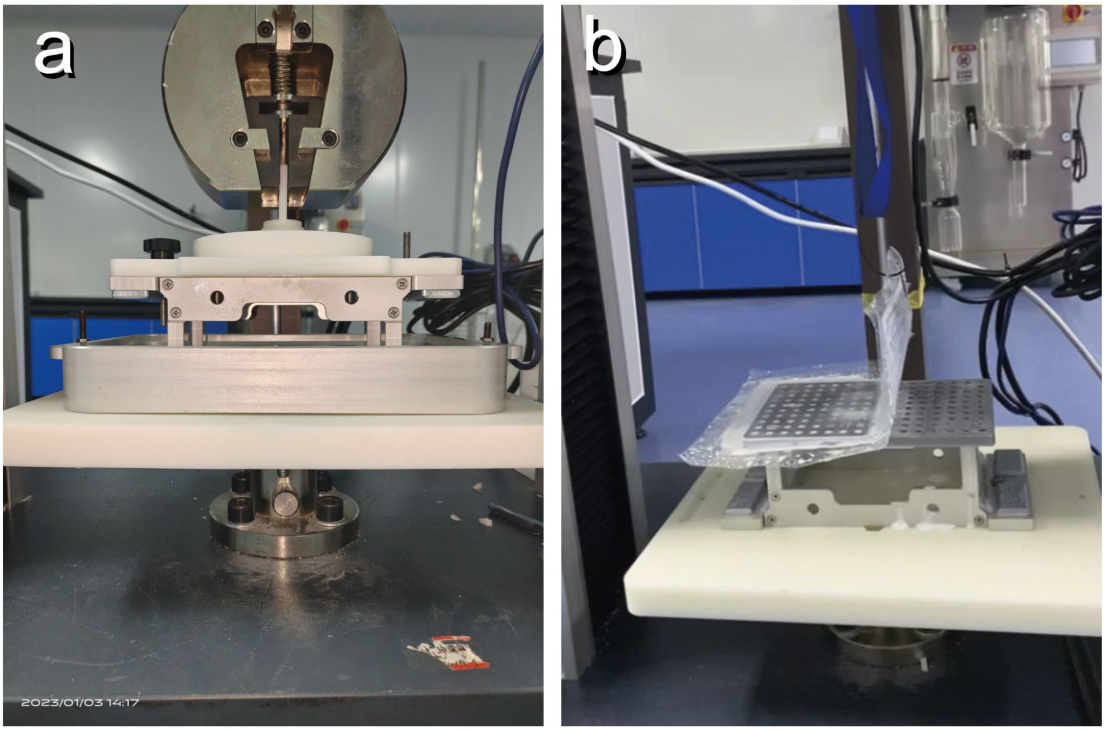

Fig. S5 Physical photos of detachment force tests setups. **a** For conventional DLP 3D printer simulation. **b** For LSVP system simulation.

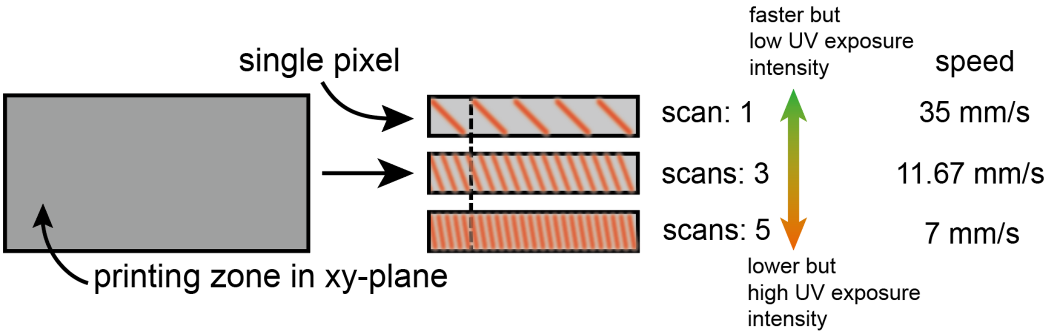

Fig. S6 Illustration of scanning strategy

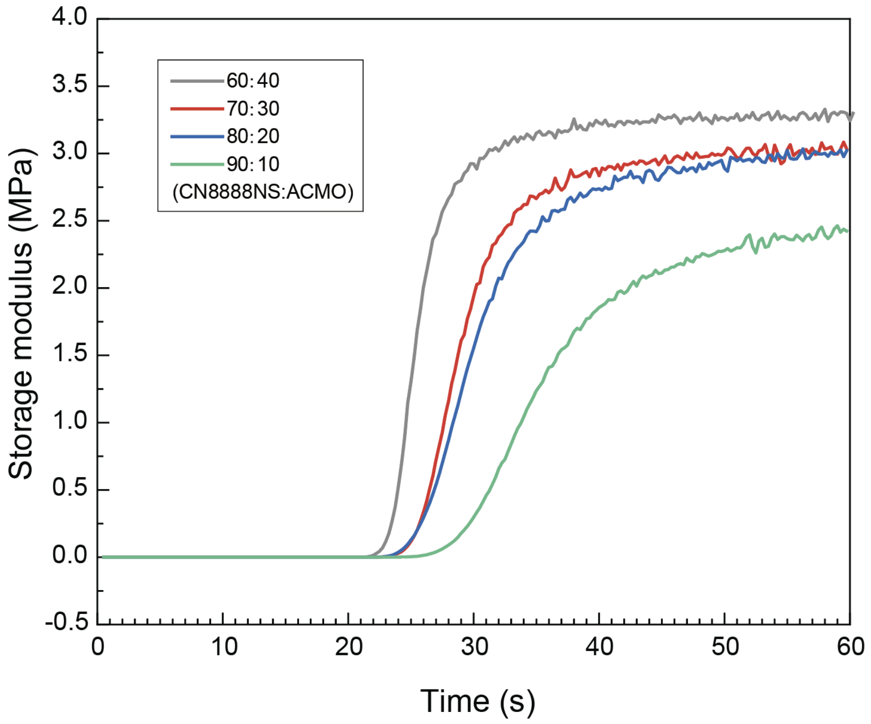

Fig. S7 Curing speed of UV curable resin with different proportions of oligomer and reactive diluent. Source data are provided as a Source Data file.

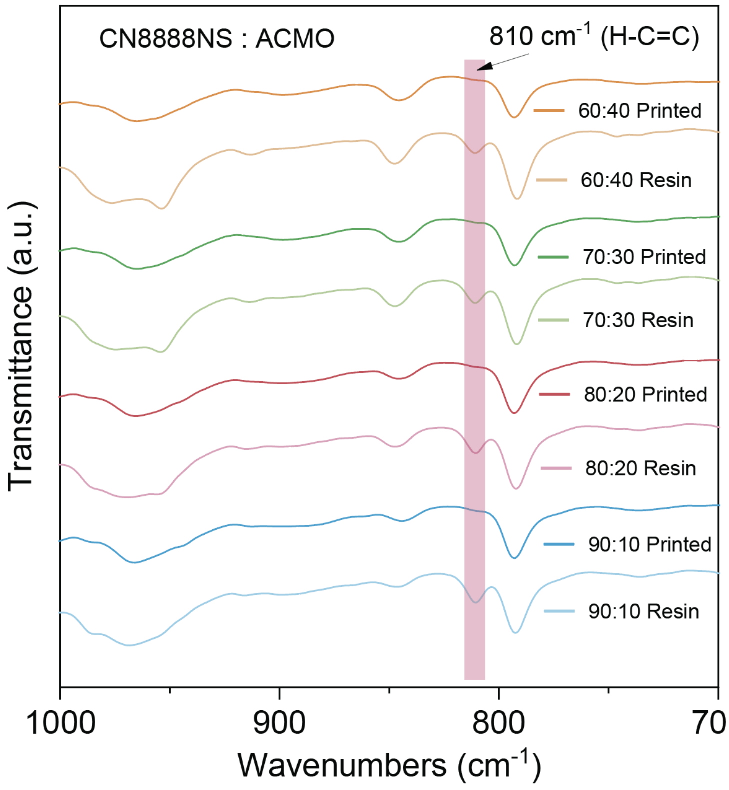

Fig. S8 FTIR spectrums of CN8888NS series samples. Source data are provided as a Source Data file.

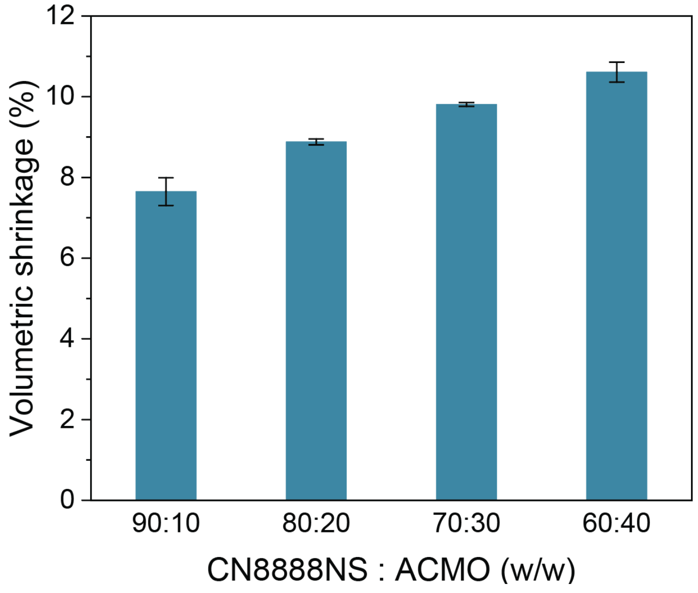

Fig. S9 **Volumetric shrinkage of CN8888NS series samples.** The error bars stand for stand errors. Source data are provided as a Source Data file.

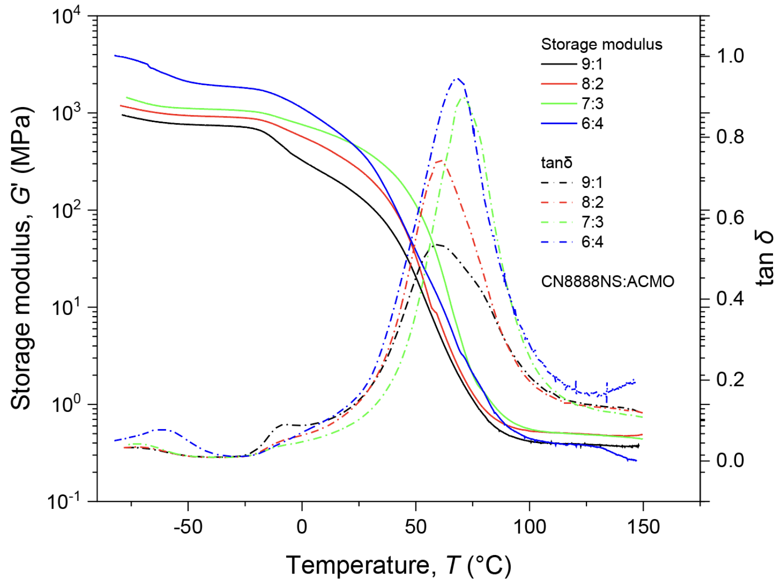

Fig. S10 **Storage modulus vs. temperature of CN8888NS series samples.** Source data are provided as a Source Data file.

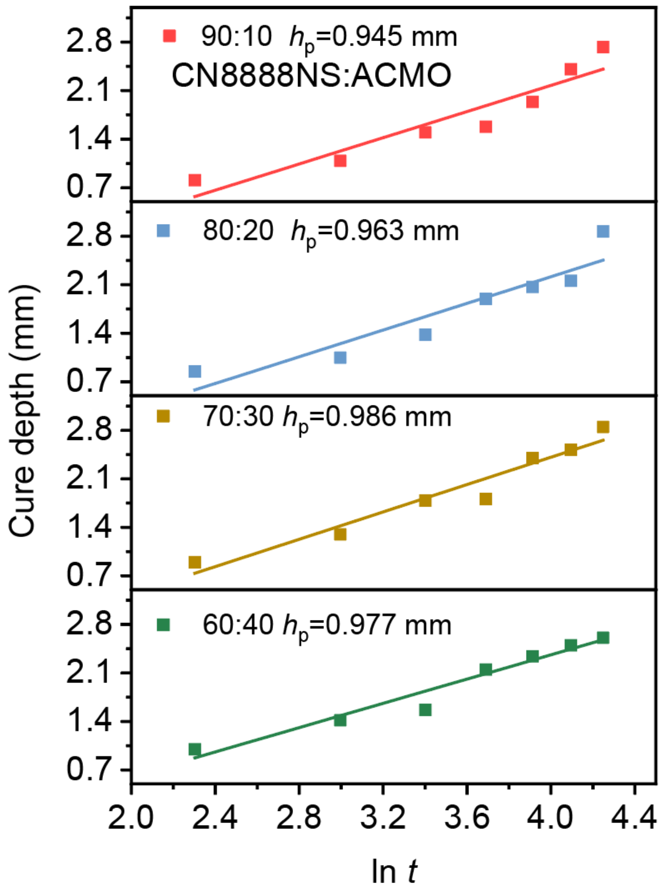

Fig. S11 Critical exposure energy and curing depth of CN8888NS series samples. Source data are provided as a Source Data file.

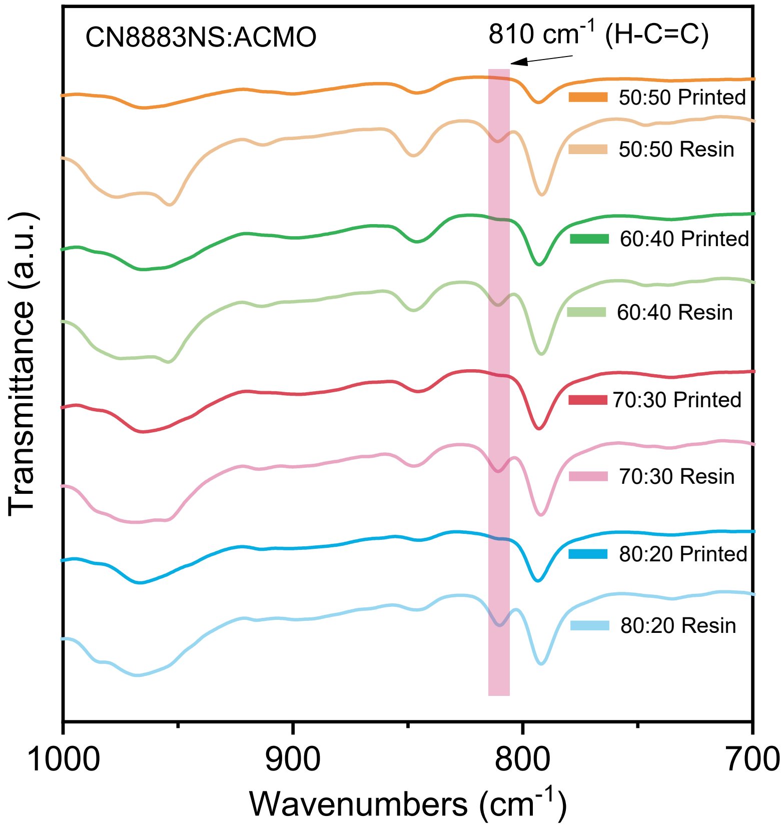

Fig. S12 FTIR spectrums of CN8883NS series samples. Source data are provided as a Source Data file.

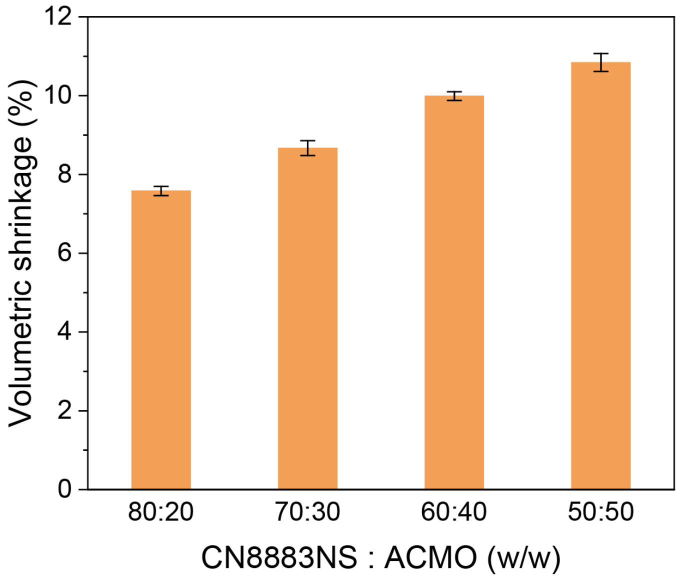

Fig. S13 Volumetric shrinkage of CN8883NS series samples. The error bars stand for stand errors. Source data are provided as a Source Data file.

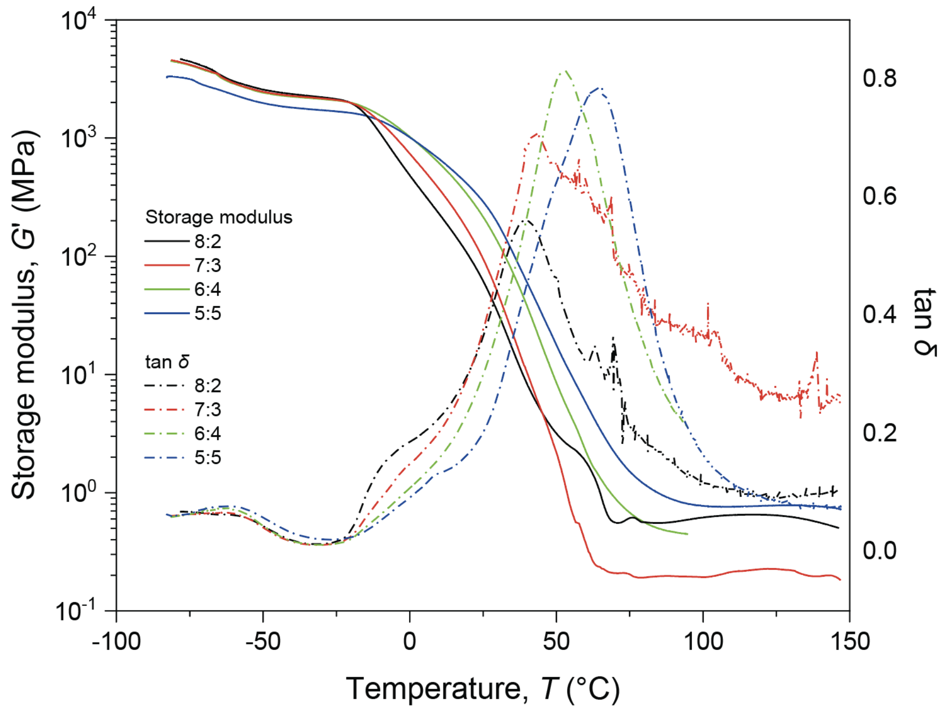

Fig. S14 Storage modulus vs. temperature of CN8883NS series samples. Source data are provided as a Source Data file.

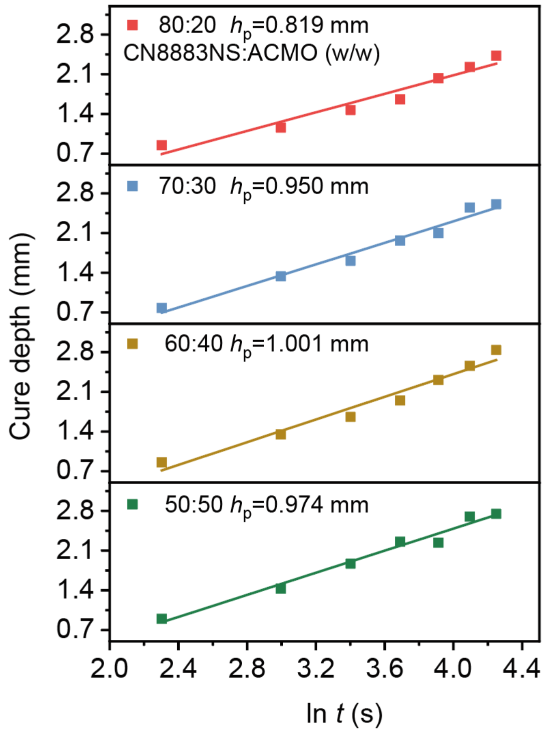

Fig. S15 Critical exposure energy and curing depth of CN8883NS series samples. Source data are provided as a Source Data file.

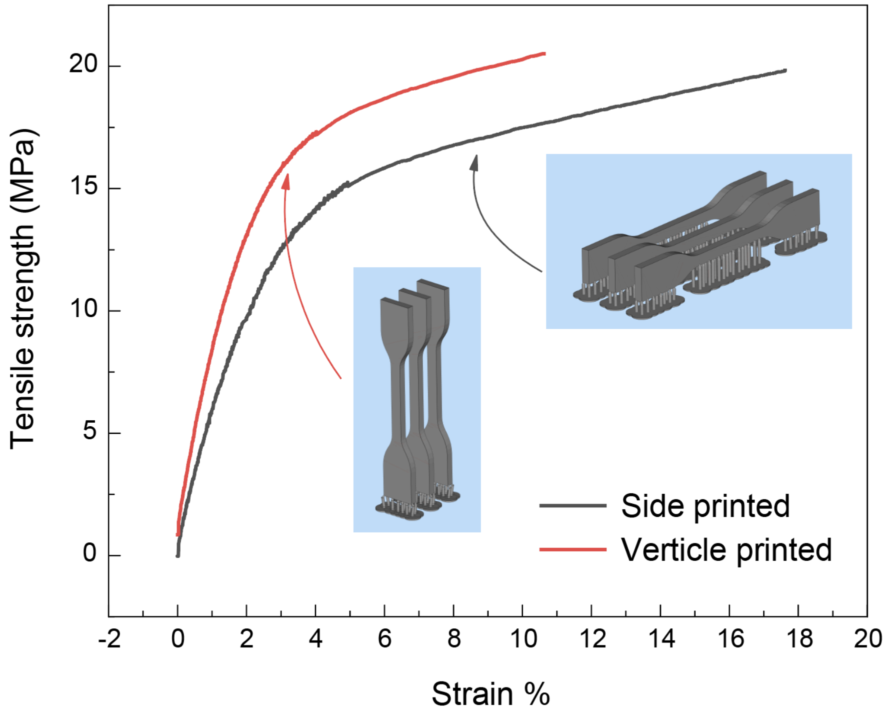

Fig. S16. Tensile strength of low viscous UV-curable resin printed in different orientation. Source data are provided as a Source Data file.

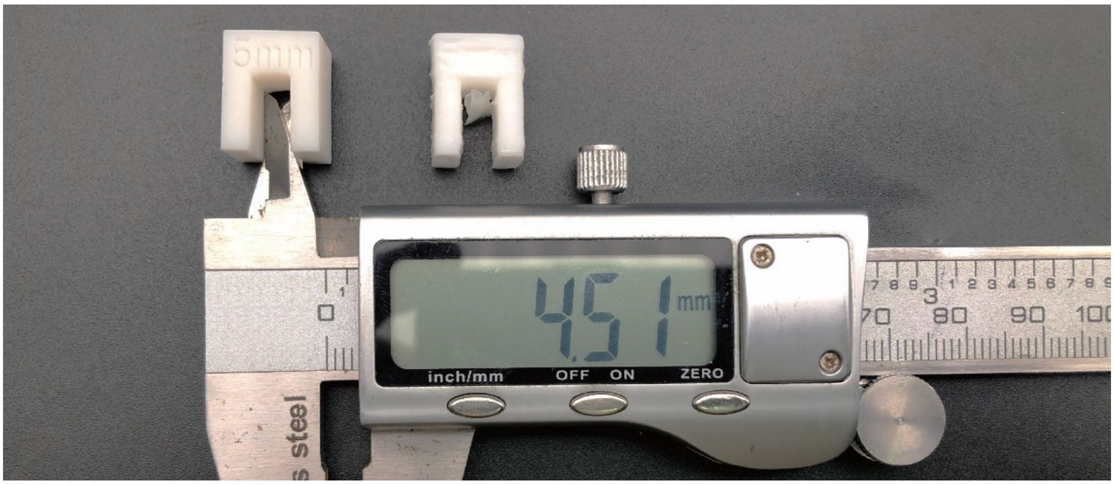

Fig. S17. Enlarged photos of span test. The left piece is printed by the LSVP system, with the carved characters clearly shown. The right piece is printed by the conventional DLP 3D printer, where the carved characters are blurred.
