# Supplementary material for: 3D printing of ultra-high viscosity resin by a linear scan-based vat photopolymerization system
Source: Nat Commun. 2023 Jul 18;14:4303. doi: 10.1038/s41467-023-39913-4 (PMC10353997; doi:10.1038/s41467-023-39913-4)
Supplement: Supplementary file 3 — Description of Additional Supplementary Files [file 41467_2023_39913_MOESM3_ESM.pdf]

Description of Additional Supplementary Files

File Name: Supplementary Movie 1
Description: Working mechanism demonstration for laser modulus in LSVP system.

File Name: Supplementary Movie 2
Description: Installation of resin tank.

File Name: Supplementary Movie 3
Description: Printing Eiffel Tower by high viscosity resin.

File Name: Supplementary Movie 4
Description: Flexural tests comparison.

File Name: Supplementary Movie 5
Description: Carving comparison between traditional UV-curable resin and high viscosity resin printed sample.

File Name: Supplementary Movie 6
Description: Printing of Eiffel Tower with commercial resin.
